# Discovery and Genomic Characterization of a Novel Phage P284 with Potential Lytic Ability Against *Agrobacterium tumefaciens*

**DOI:** 10.3390/plants14243755

**Published:** 2025-12-10

**Authors:** Orges Cara, Miloud Sabri, Khaoula Mektoubi, Angelo De Stradis, Toufic Elbeaino

**Affiliations:** 1 International Centre for Advanced Mediterranean Agronomic Studies (CIHEAM of Bari), 70010 Valenzano, Italy; org.cara@gmail.com (O.C.); miloud.sabri@uit.ac.ma (M.S.); mektoubikhaoula2099@gmail.com (K.M.); 2 Department of Soil, Plant and Food Science, University of Bari, 70126 Bari, Italy; 3 Regional Center of Nanotechnology NANOBALKAN, Academy of Sciences of Albania, Murat Toptani Avenue, 1000 Tirana, Albania; 4 Institute for Sustainable Plant Protection (IPSP), National Research Council of Italy (CNR), University of Bari, 70121 Bari, Italy; angelo.destradis@cnr.it

**Keywords:** bacteriophage, lytic activity, HTS, genome characterization, electron microscopy

## Abstract

*Agrobacterium tumefaciens* (*A. tumefaciens*), the causal agent of crown gall disease, is a major threat to crop production worldwide. In this study, a novel lytic bacteriophage, designated P284, was identified and characterized for its antibacterial potential against *A. tumefaciens*. High-throughput sequencing revealed a 44,922 bp double-stranded DNA genome (G+C content 54.3%), with 66 predicted coding sequences, none associated with virulence, lysogeny, or antibiotic resistance. Genomic and phylogenetic analyses allocated P284 within the genus *Atuphduovirus* (subfamily *Dunnvirinae*), showing 94% nucleotide sequence identity and 100% query coverage with phage PAT1, representing a distinct species. Turbidity assays revealed that P284 (MOI = 1) strongly inhibits *A. tumefaciens* growth up to 48 h, achieving a 92% reduction in bacterial density. Transmission electron microscopy confirmed rapid adsorption and host cell lysis within 30 min. In silico predictions identified three putative depolymerases with properties suitable for recombinant applications. The phage exhibited stability across a wide pH range (3–9) and temperatures from −20 to 60 °C. These findings highlight the lytic activity and environmental resilience of P284, and whether it can control crown gall disease in planta remains to be evaluated.

## 1. Introduction

Bacteriophages, or phages, are viruses that specifically infect bacteria and are considered the most abundant biological entities on Earth [1]. They are estimated to number around 10^31^ particles globally, outnumbering bacteria by at least an order of magnitude, and play a fundamental role in regulating microbial communities in virtually every environment, from soil and water to the human gut and plant rhizosphere. Phages display extraordinary diversity in morphology, genome size, and infection strategies, yet all share the defining feature of being obligate parasites that rely on bacterial hosts for replication [2]. Beyond their ecological importance, bacteriophages have profound biomedical and biotechnological relevance. Their role in controlling bacterial populations has long suggested potential as therapeutic agents, a concept known as phage therapy [3,4]. First explored in the early 20th century by Félix d’Hérelle, phage therapy is now regaining global interest due to the alarming rise in multidrug-resistant bacteria. Phages offer unique advantages over traditional antibiotics: they are highly specific to their bacterial hosts, self-amplifying at the site of infection, and generally harmless to eukaryotic cells. Modern advances in molecular biology and genomics have further enhanced the potential of phages, enabling genetic engineering to expand host range, improve stability, or design phage-derived antimicrobial proteins such as endolysins. In agriculture and food safety, phages are increasingly studied as potential eco-friendly alternatives for controlling bacterial pathogens of plants and animals, reducing contamination in food processing, and mitigating the spread of antibiotic resistance [3].

*Agrobacterium tumefaciens* is a Gram-negative, rod-shaped bacterium belonging to the subfamily *Rhizobiaceae*, widely known as the causal agent of crown gall disease in dicotyledonous plants [5]. This soil-borne pathogen was first described in the early 20th century and has since become one of the most studied plant-associated bacteria due to its remarkable ability to transfer its DNA to other organisms. The hallmark of *A. tumefaciens* is the presence of a tumor-inducing plasmid, which harbors the transfer of DNA and the virulent genes [6]. The disease caused by this pathogen, crown gall, is characterized by tumor-like swellings at the crown and root-stem junction of infected plants, although it can also appear on stems, branches, or leaves if wounds are present [7]. These galls disrupt water and nutrient transport, reducing plant vigor, stunting growth, and, in severe cases, causing plant decline. Economically, crown gall is of particular concern in horticulture and forestry, especially in grapevines, stone fruits, roses, and many ornamentals, where infection reduces both yield and quality.

In the case of *A. tumefaciens*, there are only eight characterized lytic phages reported in the literature to infect this bacterial plant pathogen: 7-7-1 [8], Atu_ph02 and Atu_ph03 [9], Atu_ph07, Atu_ph04 and Atu_ph08 [10], Milano [11] and PAT1, which was the last to be discovered and has demonstrated significant antibacterial efficacy against *A. tumefaciens* in vitro [12]. Expanding the diversity of characterized *Agrobacterium*-specific phages is therefore essential to establish reliable phage-based solutions for agriculture. In particular, the identification and functional analysis of lytic genes, including *depolymerases* and *hydrolases*, are critical for understanding infection mechanisms and for the potential development of phage-derived antimicrobial enzymes. In this study, a novel lytic phage, designated P284, was isolated from sewage water in Bari, Italy, and subjected to morphological, genomic, and functional characterization to evaluate its potential as a biocontrol agent against *A. tumefaciens.*

## 2. Results

### 2.1. Plaque Morphology and Host Range of Phage P284

**Figure 1:**
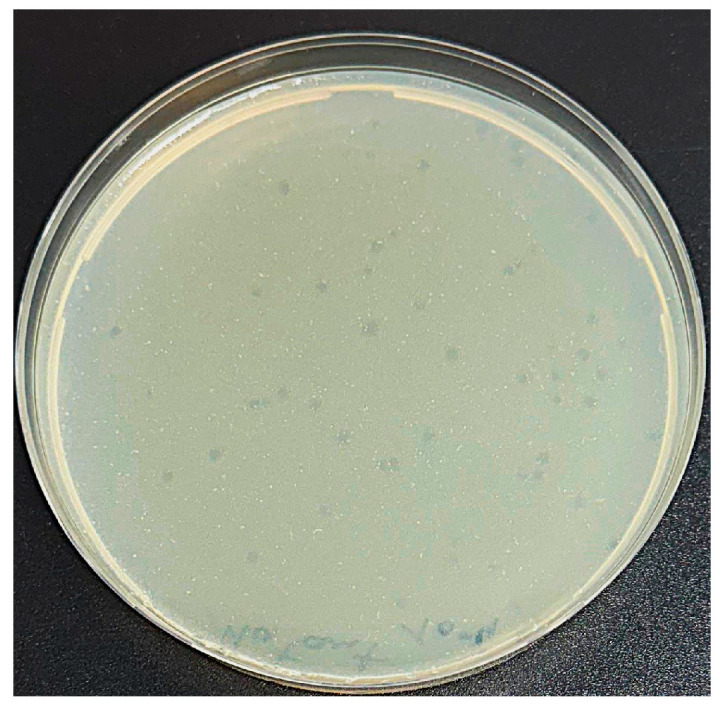
Plaque assay showing well-defined lytic plaques (~2 mm in diameter) formed by phage P284 on *A. tumefaciens* lawn.

A novel phage, hereafter designated P284, capable of infecting *A. tumefaciens*, was isolated from sewage water collected in Bari, Italy. In double agar overlay assays, P284 produced clear, circular plaques approximately 2 mm in diameter on *A. tumefaciens* BPIC 284 lawns (Figure 1). Host range analysis revealed that P284 exclusively infected *A. tumefaciens* strain BPIC 284 among all bacterial strains tested, suggesting a highly specific and narrow host range.

### 2.2. Transmission Electron Microscopy

**Figure 2:**
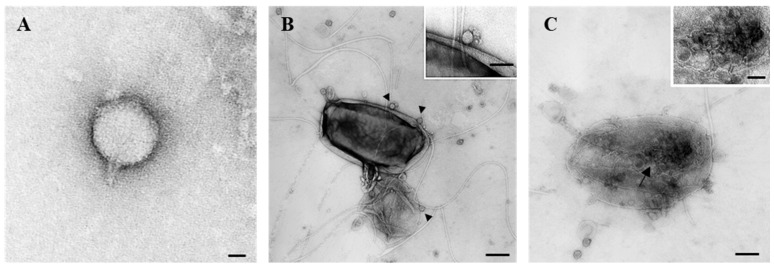
Transmission electron micrographs showing phage P284 and its’ interaction with BPIC 284 cells. (**A**) phage P284 particle with an isometric head and a short tail structure. (**B**) Phage P284 adsorption to the bacterial cell surface, with virions attached to the host envelope (arrowheads) (inset shows a magnified view of a single phage at the cell surface). (**C**) Advanced stage of infection, with numerous phage particles inside (arrow) (inset shows a magnified view of phages aggregate) and a bacterial cell undergoing structural damage and lysis. Bars: (**A**) 25 nm; (**B**) 100 nm, inset 50 nm; (**C**) 100 nm, inset 50 nm.

Transmission electron microscopy (TEM) analysis revealed that phage P284 displayed a C1 podovirus morphotype, with an isometric head measuring 50 nm in diameter, with a short, non-contractile tail of approximately 15 nm (Figure 2A). Ultrastructural examination further demonstrated that adsorption of P284 particles onto the surface of BPIC284 cells occurred 30 min post-infection (pi) (Figure 2B). At this stage, bacterial cell lysis was evident, accompanied by the release of numerous progeny phage particles (Figure 2C).

### 2.3. Turbidity Assay

**Figure 3:**
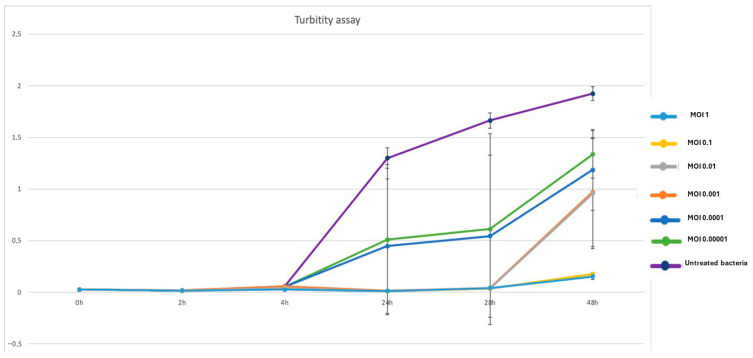
Turbidity assay showing the inhibitory effect of P284 treatment on BPIC284 growth at different MOI. Optical density (OD_600_) was monitored over 48 h. All phage-treated samples exhibited a marked suppression of bacterial growth compared to the untreated control, with the strongest inhibition observed at higher MOIs (1 and 0.1). Error bars represent standard deviations from three independent replicates.

Phage P284 exhibited a clear inhibitory effect on *A. tumefaciens* BPIC284 growth across all tested multiplicities of infection (MOIs) (Figure 3). At the start of the assay, all cultures showed comparable baseline optical densities (OD_600_ ≈ 0.03). A marked suppression of bacterial growth was observed in phage-treated samples from 4 h onward, particularly at higher MOIs (1 and 0.1), where the OD_600_ values remained near zero throughout the 48 h period. In contrast, untreated bacteria displayed a continuous increase in turbidity, reaching OD_600_ ≈ 2.0 at 48 h. Intermediate MOIs (0.01–0.0001) resulted in partial inhibition, with gradual bacterial regrowth after 24–48 h, suggesting the emergence of resistant or phage-tolerant subpopulations. Overall, these results demonstrate that P284 efficiently suppresses *A. tumefaciens* proliferation in a dose-dependent manner, with sustained inhibition at higher phage-to-bacterium ratios and delayed regrowth at lower MOIs.

### 2.4. Complete Genome Sequence and Phylogenetic Analysis of P284

**Figure 4:**
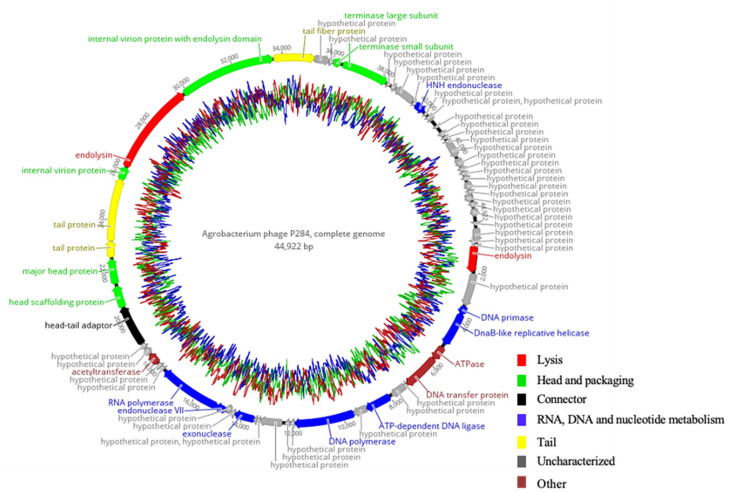
Circular genome map of P284 generated with Geneious Prime^®^ 2025.2.2. The inner rings display the GC content, while predicted coding sequences annotated by Pharokka are representedas colored arrows, with arrow colors indicating their functional categories.

P284 revealed a double-stranded DNA genome structure, composed of 44,922 bp in length. The genome was shown to encode 66 predicted coding sequences (CDSs), of which 23 CDSs (34.9%) were assigned to specific functions, while 43 (65.1%) were of unknown functions. These annotated genes are associated with DNA replication and regulation, DNA packaging, structural proteins, and host cell lysis, as highlighted in the genomic map (Figure 4). No tRNA-encoding genes were identified using Prokka and Geneious analyses. Furthermore, comprehensive genome evaluation (CGE) analysis confirmed the absence of genes linked to antibiotic resistance, lysogeny, toxins, or other virulence factors, supporting the suitability of P284 as a candidate biocontrol agent.

**Figure 5:**
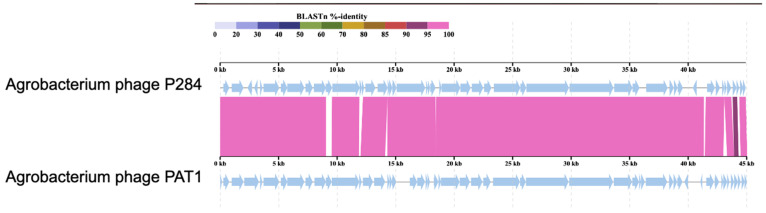
Comparative genomic alignments between P284 and PAT1 generated using ViPTree. The colored vertical blocks between the genomes indicate levels of nucleotide similarity. Arrows indicate ORFs.

Comparative genomic analysis revealed that P284 shares the highest nucleotide sequence identity with *Agrobacterium* phages PAT1 (94%; accession PQ082932.1) (Figure 5), Atu_ph02 (78.7%; accession NC_047845) and Atu_ph03 (78.5%; accession NC_047846), both members of the genus *Atuphduovirus* (subfamily *Dunnvirinae*), known to infect *A. tumefaciens*, which, according to ICTV demarcation criteria (95% species threshold), confirms that P284 represents a novel species within the same taxonomic order, *Autographivirales*.

**Figure 6:**
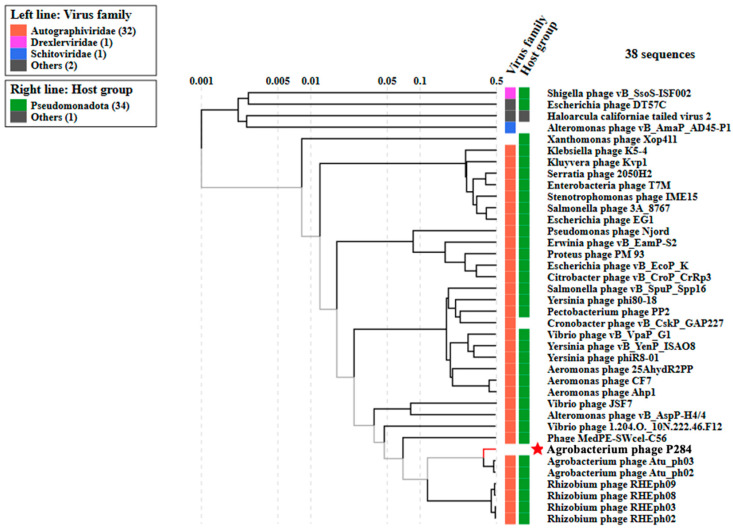
Proteomic tree of P284, generated by ViPTree (version 4.0) based on genome-wide sequence similarities computed by tBLASTx, showing a close relationship with *Agrobacterium* phage ph03 and ph02. This phylogenetic tree was generated on 30 September 2025, and the VipTree database had not yet been updated to reflect the most recent taxonomic ratifications. Note that in 2025, the family *Autographiviridae* was elevated to the rank of order and renamed *Autographivirales*. The star indicates the phage P284 identified in this study.

Furthermore, phylogenetic analysis based on whole-proteome comparisons, generated using ViPTree, corroborated the genomic analyses and positioned P284 alongside other *Agrobacterium* phages (Figure 6). The complete genome sequence of P284 was deposited in NCBI GenBank under the accession number PX606436.

### 2.5. In Silico Identification of Putative Antibiofilm-Associated Depolymerases in P284

Computational identification of depolymerase-encoding genes in the genome of P284 was performed using DePP [13], which predicted three candidate proteins (P35, P38, and P39) with high probabilities of depolymerase activity (84.4–91.7%). Their predicted structural and physicochemical properties are summarized in Table 1. These proteins range from 521 to 1254 amino acids (57–137 kDa) and display mildly acidic theoretical pI values (4.9–5.7). ProtParam analysis classified all three as hydrophilic (negative GRAVY scores) and stable (instability index < 40), with half-lives exceeding 10 h in *E. coli*, supporting their potential suitability for recombinant expression. Further HHpred analysis revealed that P35 and P39 share homology with bacteriophage tail-associated structural proteins but lack detectable similarity to characterized depolymerases, whereas P38 was assigned with 100% probability to the peptidoglycan hydrolase gp16 family (Table 1). Peptidoglycan hydrolase gp16 is a phage-encoded lytic enzyme that hydrolyzes bonds within the bacterial peptidoglycan layer, facilitating localized cell wall degradation [14]. This activity is crucial during the initial stage of infection, where gp16 assists in penetrating the bacterial envelope and promotes efficient ejection of phage genomic DNA into the cytoplasm. Tail-associated gp16 hydrolases are often integrated into the viral injection apparatus, acting synergistically with mechanical forces of the tail to breach the cell wall barrier [15].

### 2.6. Predicted Lysis Modules of P284

Genomic annotation of P284 using Pharokka, followed by structural validation through HHpred analysis, enabled prediction of two putative lysis-associated proteins (P5 and P37) (Table 2). Both were annotated as endolysins, with lengths of 343 and 1214 amino acids, respectively. P5 (36.9 kDa) displayed a high aliphatic index (84.02), low instability index (16.13), and a predicted 10 h half-life in *E. coli*, with HHpred assigning a 99% probability match to a characterized endolysin. P37 (131.5 kDa), though larger and less stable (instability index 33.01), also showed a 10 h half-life and strong HHpred similarity (99% probability) to internal virion protein gp15. The physicochemical profiles highlight differences in size, stability, and hydropathicity between the two proteins, supporting their putative role in the lysis module of P284.

### 2.7. Temperature and pH Stability

**Figure 7:**
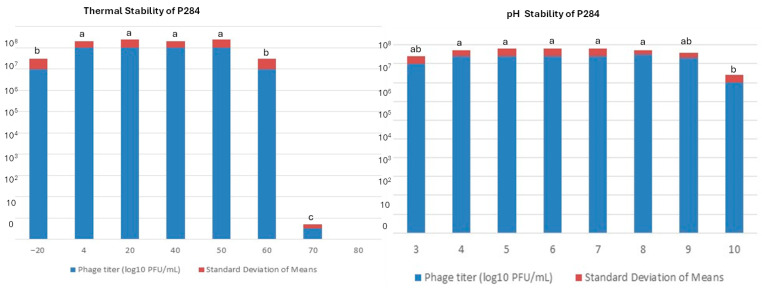
Thermal and pH stability of phage P284. Phage titers following exposure to various temperatures for 1 h. Phage infection after 24 h incubation at different pH values. Titers were quantified using the double agar overlay assay. Standard deviations are calculated from three independent replicates. a, b, ab and c: statistical significance groups.

The stability of P284 under different thermal and pH conditions was evaluated. Thermal assays showed that P284 maintains high infectivity from 4 °C to 50 °C, with modest but statistically significant reductions at −20 °C and 60 °C, and an almost complete loss of activity at 70 °C, indicating a thermal inactivation threshold (Figure 7). Similarly, pH assays demonstrated that P284 remains stable between pH 3 and pH 9, with no significant differences across these treatments, while a marked decrease in titer was observed at pH 10 (Figure 7).

These findings imply that the P284 exhibited significant tolerance to extreme physicochemical environments, thereby reinforcing its prospective application in diverse biotechnological and agricultural contexts.

## 3. Discussion

The isolation and characterization of phage P284 provides new insights into the potential application of bacteriophages for the control of *A. tumefaciens*, a pathogen of major concern in horticulture and forestry due to its ability to induce crown gall disease. The strict lytic activity of P284, coupled with its narrow host range, restricted to *A. tumefaciens* BPIC284, positions this phage as a double-edged tool for the biological control of crown gall disease. Its high specificity could be considered advantageous because it minimizes off-target effects and preserves beneficial or neutral microorganisms within the plant microbiome, an increasingly important criterion for environmentally responsible plant disease management. However, such narrow specificity may limit its applicability against *A. tumefaciens* strains genetically distant from BPIC284. Whether this represents a practical limit remains to be determined through broader host-range assays, including strains not evaluated in the present study. Importantly, only a very limited number of strictly lytic phages active against *A. tumefaciens* have been reported in the literature [16]. This scarcity underscores the relevance of P284 as a valuable addition to the currently small phage group available for crown gall control.

In phage-based biocontrol strategies, a key concern is the potential risk associated with releasing phages whose genomes contain numerous hypothetical proteins of unknown function, as these may lead to unforeseen interactions with non-target or beneficial environmental bacteria. For P284, however, this risk is substantially lesser, whereas unlike many newly discovered phages whose genomes contain a high proportion of uncharacterized ORFs, a high number (23 ORFs) of the annotated gene products in P284 encode proteins with known and well-described biological functions, commonly found among lytic phages. This substantially lowers the likelihood of unexpected ecological effects or cryptic interactions within microbial communities. Additional genomic features, including its streamlined genome architecture, absence of tRNAs, and lack of virulence or antibiotic resistance genes, further align P284 with established safety criteria for biocontrol phages [17].

The absence of tRNA genes in P284 is also noteworthy. In small-genome phages, the presence of tRNAs is often associated with an expanded host range or enhanced ability to infect multiple bacterial species [18]. In contrast, the lack of tRNAs in P284 is consistent with its high specificity and narrow host range. From a practical perspective, and particularly in regulatory and biosafety evaluations, phages that do not encode tRNAs are generally preferred because they are considered more host-specific and less prone to rapid evolutionary adaptation beyond the target pathogen.

The turbidity assays indicated that P284 effectively suppressed *A. tumefaciens* growth for up to 24 h, though regrowth was observed after prolonged incubation. This outcome may reflect the emergence of resistant bacterial subpopulations, a phenomenon commonly encountered in phage-host systems. In this study, our experimental focus was directed toward evaluating the lytic performance of P284 across a range of MOIs under conditions relevant to biocontrol applications. However, a classical one-step growth curve was not performed; therefore, specific replication parameters such as latent period and burst size could not be determined. In this context, the turbidity-based killing assay conducted across a range of MOIs indicates rapid and effective lytic activity of P284 against *A. tumefaciens*. Future work will include a full one-step growth curve analysis to complement the current findings and further elucidate the replication dynamics of P284.

While laboratory assays provide solid evidence on the lytic activity of P284, further evaluations under greenhouse and field conditions are required to confirm efficacy, persistence, and compatibility with existing crop protection practices. Multi-season trials, assessment of phage cocktail strategies, and formulation development will be essential steps toward translating P284 into a practical tool for sustainable management of crown gall disease.

## 4. Materials and Methods

### 4.1. Bacterial Strains and Culture Conditions

The bacterial strains used throughout this study (Table 3) were grown at 28 °C either in liquid yeast extract peptone glucose broth (YPG) (5 g/L yeast extract, 5 g/L peptone and 10 g/L glucose) or on yeast extract peptone glucose agar (YPGA, i.e., YPG supplemented with 1.5% agar). For long-term storage, the strain was stored at −80 °C in 25% glycerol prepared in YPG. The strains included both phytopathogenic and beneficial bacteria to evaluate the specificity and safety of isolated phages.

### 4.2. Bacteriophage Isolation, Purification, and Titration

Phages were isolated from a sewage water sample collected in January 2024 from the untreated influx point at the wastewater processing station of Bari (south of Italy). One liter of sewage water underwent initial filtration using Grade 1 filter paper (Whatman, Maidstone, UK) to remove larger particles, followed by filtration through a nylon Acrodisc^®^ syringe filter (Merck, Rome, Italy) with 0.22 μm pores to remove cellular debris. The filtrate was then centrifuged at 108,763× *g* (Rotor J50.2 Ti, Beckmann, Brea, CA, USA) for 1 h at 4 °C to concentrate phage particles. The concentrated phages were suspended in phage buffer (100 mM Tris-HCl (pH 7.6); 10 mM MgCl_2_; 100 mM NaCl; and 10 mM MgSO_4_). This suspension was then mixed with an overnight culture of strains (Table 3) in YPG medium, incubated overnight at 25 °C, and then the supernatant was filtered (0.22-μm filter) and stored at 4 °C. Phages were isolated and purified from the filtrate via the standard double agar overlay method [19]. A single clear plaque-forming unit was transferred into 1 mL of phage buffer, and this process was repeated three times to ensure the isolation of a single phage. To obtain high phage titer, 1 mL of *A. tumefaciens* strain BPIC 284 (OD_600_ = 0.2) was inoculated in 500 mL YPG medium, and 1 mL of purified phage was added, and the mix was then incubated for up to 24 h at 25 °C. Amplified phages were filtered through 0.22-μm filters, concentrated by high-speed centrifugation at 108,763× *g* for 2.5 h, resuspended in 2 mL of phage buffer, and stored at 4 °C for further analysis. The phage titer was determined through a double-layer assay.

### 4.3. Turbidity Assay

The antibacterial activity of phages against bacterial strains was evaluated using a turbidity assay based on OD measurements. A fresh overnight culture of strains was adjusted to an initial OD_600_ of ~0.2 in nutrient broth [20]. For the infection assay, 100 μL of bacterial suspension was mixed with 100 μL of purified phage suspension or their serial dilutions (10^−1^ to 10^−5^). Negative controls consisted of 100 μL sterile water mixed with 100 μL of bacterial suspension. The mixtures were placed in 1.5 mL sterile tubes and incubated at 28 °C in a Ski4 shaking incubator (120 rpm). Bacterial growth was assessed by measuring OD_600_ at 2, 4, 24, 28, and 48 h using a UV-1800 Shimadzu spectrophotometer. All measurements were blank corrected against sterile medium [21]. The collected data were used to evaluate growth-inhibited kinetics, including the onset of suppression, the duration of inhibition, and potential regrowth events indicating the emergence of resistant bacterial subpopulations. This experiment was performed in triplicate.

### 4.4. Transmission Electron Microscopy and Metadata

Phage growth dynamics were examined by exposing the bacteria culture (BPIC284) to the phage (P284) at MOI = 1 under room temperature conditions. Aliquots were collected at 15-, 30-, 60-, and 120 min post-infection and analyzed by transmission electron microscopy (TEM; FEI MORGAGNI 282D, Thermo Fisher Scientific, Waltham, MA, USA) using the dip method according to Milne [22]. For this, carbon-coated copper/rhodium grids were incubated for 2 min with either phage-infected or untreated bacterial suspensions, followed by rinsing with 200 μL of distilled water. Negative staining was performed by immersing the grids in 200 μL of 0.5% (*w*/*v*) UA-Zero EM stain (Agar-Scientific Ltd., Catcliffe, Rotherham, UK), and visualization was carried out at an accelerating voltage of 80 kV.

The phages’ metadata, head diameter and tail length were extracted from the electron microscope micrographs using Adobe Photoshop software (version 6.0.1) for dimensional measurements and analyzed using standard Excel statistical formulas; counts were determined on a sample of 50 units per population.

### 4.5. DNA Extraction, Whole Genome Sequencing, and Bioinformatic Analysis

Phage DNA was extracted using a CTAB and phenol-chloroform protocol. Briefly, concentrated phage lysates were treated with DNase and RNase to remove host nucleic acids, followed by proteinase K digestion in the presence of SDS (10%) and EDTA (25 mM) to lyse the capsid [23]. The lysate was mixed with CTAB buffer and incubated at 65 °C, then extracted with phenol: chloroform to remove proteins and impurities. DNA was precipitated using sodium acetate and cold ethanol, washed with 70% ethanol, and resuspended in nuclease-free water for sequencing. The extracted DNA was quantified using the NanoDrop^TM^ One/OneC Microvolume UV-Vis Spectrophotometer (ThermoFisher Scientific, Waltham, MA, USA). Subsequently, 500 ng of purified genomic DNA was sent for Illumina sequencing (2 × 150 bp paired-end mode) (Eurofins Genomics, Cologne, Germany). The reads were quality-checked, trimmed, and de novo assembled using the Tadpole tool with different k-mers (Geneious Prime 2025.2.2; San Diego, CA, USA). Open reading frames (ORFs) were functionally annotated using the Galaxy Phage platform with the ‘Pharokka’ tool [24]. In addition, putative antibiofilm-associated depolymerases were predicted using De-Polymerase Predictor (DePP) [13], followed by structural and physicochemical characterization with ProtParam [25] and HHpred [26]. Likewise, HHpred analysis was also employed to refine functional annotation of lysis-associated proteins, enabling the prediction of the putative lytic genes. The complete genome sequence of the sequenced phage was deposited at GenBank, and a circular map of the genome and a phylogenetic tree were constructed using ViPTree [27].

### 4.6. Temperature and pH Stability

Thermal stability of the phage was evaluated by incubating aliquots of phage suspensions (~1 × 10^8^ PFU/mL) for 24 h at −20, 4, 20, 40, 50, 60, and 70 °C, with each treatment performed in three independent replicates. Residual infection was determined by serial dilution and quantification using the double-layer agar plaque assay on bacterial strains. To evaluate pH stability, phage suspensions were diluted 1:10 in sterile phage buffer and adjusted to pH 3–10 with 1 M HCl or 1 M NaOH. Samples were incubated at 28 °C for 24 h, after which surviving phages were enumerated by plaque assay.

### 4.7. Statistical Data Analysis

Statistical analyses were performed using RStudio (version 4.5.1). Data normality was assessed using the Shapiro–Wilk test, which revealed a significant deviation from normality. Consequently, the nonparametric Kruskal–Wallis test was applied to compare treatments. Post hoc pairwise comparisons were carried out using Dunn’s test with Bonferroni correction to adjust for multiple comparisons. Statistically significant groupings were determined using the multcompLetters function. Mean values and standard deviations were calculated for each treatment for graphical representation.

## 5. Conclusions

This study reports the discovery of a novel phage species (P284), which emerges as a promising candidate for the biocontrol of *A. tumefaciens* due to its strict lytic nature, genetic safety features, and effective suppression of bacterial growth. The narrow host range, while limiting activity to a subset of *A. tumefaciens* strains, enhances ecological safety by minimizing unintended effects on beneficial microbiota. Broader host-range testing with geographically and genetically diverse *A. tumefaciens* isolates will clarify its operational spectrum and guide potential phage-cocktail formulations. Future work should validate its performance in greenhouse and field settings and explore integration into phage-based or combined biocontrol strategies for sustainable crown gall management.

## Figures and Tables

**Table 1:** Predicted phage-encoded depolymerases and their physicochemical and structural properties derived from DePP, ProtParam, and HHpred analyses.

Code	Function (Pharokka)	Depolymerase Prediction (%) (DePP)	Length (aa)	MW (kDa)	pI	Instability Index	Aliphatic Index	GRAVY	Half-Life (*E. coli*)	HHpred Top Hit (Probability, Aligned Cols)
P35	Tail protein	91.7	823	91.3	5.1	33.18	77.08	−0.228	10 h	Tail tubular protein B (100%, 765)
P38	Internal virion protein with endolysin domain	84.4	1254	137.3	5.7	35.28	71.40	−0.464	10 h	Peptidoglycan hydrolase gp16 (100%, 938)
P39	Tail fiber protein	84.9	521	55.0	4.9	29.31	68.35	−0.268	10 h	Tail fiber protein (99.61%, 140)

**Table 2:** Predicted lysis-associated proteins of P284 and their physicochemical properties.

Code	Function (Pharokka)	Length (aa)	MW (kDa)	pI	Instability Index	Aliphatic Index	GRAVY	Half-Life (*E. coli*)	HHpred Top Hit (Probability, Aligned Cols)
P5	Endolysin	343	36.9	9.4	16.13	84.02	−0.067	10 h	Endolysin (99%, 206)
P37	Endolysin	1214	131.5	5.7	33.01	67.53	−0.604	10 h	Spore cortex-lytic enzyme (99%, 222)

**Table 3:** Bacterial strains used for phage host range determination.

Species	Strain ID	Host Plant	Origin
*Bacillus siamensis*	C36 *	*Brassica oleracea* var. *botrytis*	Italy
*Bacillus subtilis*	C70 *	*Brassica oleracea* var. *botrytis*	Italy
*Pseudomonas graminis*	C23 *	*Brassica oleracea* var. *botrytis*	Italy
*Pantoea agglomerans*	C6 *	*Brassica oleracea* var. *botrytis*	Italy
*Pseudomonas fulva*	C29 *	*Brassica oleracea* var. *botrytis*	Italy
*Pseudomonas putida*	B14 *	*Brassica oleracea* var. *italica*	Italy
*Pseudomonas hunanensis*	B10 *	*Brassica oleracea* var *. italica*	Italy
*Lactococcus lactis* subsp. *lactis*	ATCC 11454	-	USA
*Leuconostoc mesenteroides*	MS4	water	Morocco
*Pseudomonas fluorescens*	CFBP 2392	*Phaseolus vulgaris*	France
*Agrobacterium vitis*	BPIC 1009	*Vitis vinifera*	Greece
*Agrobacterium vitis*	CFBP 2738	*Vitis vinifera*	Greece
*Agrobacterium tumefaciens*	BPIC 310	*Pyrus amygdaliformis*	Greece
*Agrobacterium tumefaciens*	BPIC 284	*Prunus dulcis*	Greece
*Agrobacterium tumefaciens*	BPIC 139	*Vitis vinifera*	Greece
*Agrobacterium tumefaciens*	YD 5660-2007	*Prunus dulcis*	Greece
*Agrobacterium tumefaciens*	YD 5156-2018	*Prunus domestica*	Greece
*Agrobacterium tumefaciens*	CFBP 5770	*Prunus persica*	Australia
*Agrobacterium rubi*	CFBP 5521	*Rubus* sp.	Germany
*Agrobacterium larrymoorei*	CFBP 5473	*Ficus benjamina*	USA

* Collection of beneficial bacteria at the Bacteriology and Phage Biocontrol laboratories, CHEAM Bari, Italy. CFBP: French Collection of Phytopathogenic Bacteria, Angers, France. YD and BPIC: Collection of bacterial strains of Benaki Phytopathological Institute (BPIC, Greece). ATCC: American Type Culture Collection, Manassas, VA, USA.

## Data Availability

Accession numbers of the sequences reported in the study is publicly available at NCBI, and data generated in the study are included in this published paper.
